# Loop-Mediated Isothermal Amplification as an Alternative to Conventional Smear Microscopy in Pulmonary Tuberculosis

**DOI:** 10.7759/cureus.117122

**Published:** 2026-09-29

**Authors:** Shivani Sharma, Parakriti Gupta, Anand Singhal, Ivneet Kour, Harpreet Kaur, Varsha Gupta, Lipika Singhal

**Affiliations:** 1 Microbiology, Government Medical College and Hospital, Sector 32, Chandigarh, IND; 2 Microbiology, All India Institute of Medical Sciences, Vijaypur, IND; 3 Internal Medicine, MM Institute of Medical Sciences and Research, Mullana, IND

**Keywords:** crs, genexpert, pulmonary tuberculosis, rapid diagnosis, tb-lamp

## Abstract

Background: Timely and accurate diagnosis of pulmonary tuberculosis (PTB) is a cornerstone of optimal disease management. Loop-mediated isothermal amplification (LAMP) is a simple yet potentially useful novel diagnostic tool for TB diagnosis that requires minimal resources. This pilot study was conducted to evaluate the diagnostic performance of TB-LAMP in diagnosing PTB in adults.

Methods: A prospective observational study was conducted, enrolling 100 adults with symptoms suggestive of PTB. Sputum samples were collected and subjected to smear microscopy, culture, GeneXpert, and TB-LAMP in parallel. A composite reference standard (CRS) involving clinical, microbiological, and radiological parameters was used for comparison. Data were analyzed using SPSS version 26.0. The sensitivity, specificity, positive predictive value, and negative predictive value of the TB-LAMP assay and other diagnostic tests were calculated.

Results: This study demonstrated a sensitivity and specificity of TB-LAMP compared with the CRS of 74.36% and 91.80%, respectively. TB-LAMP helped identify cases that conventional techniques missed, highlighting its potential for early TB detection.

Conclusion: This study demonstrates the feasibility and diagnostic potential of TB-LAMP for detecting PTB in adults. Its sensitivity and specificity underscore its potential role in accurate diagnosis. However, further validation in a larger and more diverse cohort is required to support its integration into routine TB diagnostic algorithms.

## Introduction

Tuberculosis (TB), a formidable global health challenge caused by *Mycobacterium tuberculosis*, has again resurfaced as the leading cause of death from any single infectious agent [1]. Nearly 87% of the global TB burden is attributed to 30 high-burden countries, with India contributing 25% of the total cases [1]. According to the annual TB report of India 2024, a total of 25.5 lakh cases of TB were notified [2]. Conventional diagnostic assays, such as smear microscopy and culture, are time-consuming and often less sensitive, while molecular assays are costlier, require extensive training and expensive equipment, and have high maintenance costs [3]. Early detection is essential for reducing disease transmission and improving treatment outcomes in pulmonary tuberculosis (PTB). Accurate diagnosis remains challenging, especially in patients with paucibacillary disease [4].

Conventional Ziehl-Neelsen (ZN) smear microscopy is quick and affordable, but it requires a bacillary load of roughly 5,000 to 10,000 bacilli/mL of sputum for accurate identification and has a variable sensitivity ranging from 20% to 80% [5]. Because of its high specificity and low detection limit (10 to 100 viable bacilli/mL), culture is still the gold standard in microbiology. However, depending on the culture system, it takes two to eight weeks to provide results. Molecular techniques, like GeneXpert MTB/RIF (GeneXpert), have a limit of detection of about 131 bacilli/mL, show an overall sensitivity of 85%-90% in PTB, with specificity surpassing 98%, and enable quick diagnosis within two hours. The World Health Organization (WHO)-approved GeneXpert has significantly improved TB diagnosis and provides results within two hours [6]. GeneXpert requires a constant electricity supply, equipment investment, and ongoing maintenance.

In contrast, tuberculosis loop-mediated isothermal amplification (TB-LAMP), a novel manual molecular TB detection method, offers a rapid (less than two hours) and resource-efficient process, requiring minimal instrumentation and providing a visually detectable fluorescent result [7]. However, the diagnostic accuracy of TB-LAMP compared with established microbiological methods needs to be further evaluated. The primary objective of this study was to evaluate the diagnostic accuracy of TB-LAMP for diagnosing PTB in adults using a composite reference standard (CRS) that incorporated clinical, radiological, and microbiological criteria. Secondary objectives were to compare the diagnostic performance of TB-LAMP with smear microscopy, culture, and GeneXpert.

## Materials and methods

This was a prospective, observational pilot study conducted over a period of six months (July 2023 to December 2023) in the Department of Microbiology at our tertiary care center. As this study was designed as a pilot diagnostic accuracy study, no formal hypothesis-based sample size calculation was performed. A pragmatic sample of 100 consecutive eligible adults presenting during the six-month study period was included according to the predefined inclusion and exclusion criteria.

Inclusion and exclusion criteria

Adults (≥18 years) with clinical suspicion of PTB who provided a suitable respiratory sample for microbiological analysis and signed informed consent were included. The presence of one or more symptoms suggestive of PTB, such as cough of ≥2 weeks' duration, fever of ≥2 weeks' duration, unexplained or significant weight loss, hemoptysis, or chest radiographic abnormalities suggestive of PTB, was the basis for clinical suspicion of PTB. Individuals under the age of 18 years, those who did not provide consent, and those with insufficient or otherwise inappropriate specimens were excluded. A sufficient sputum specimen was required to allow allocation of 0.05 mL for ZN staining, 1 mL for culture, 1 mL for GeneXpert, and 0.06 mL for TB-LAMP. Specimens that did not provide adequate volume for these planned investigations were considered insufficient. Saliva-dominant or otherwise non-representative respiratory specimens were considered inappropriate and were excluded.

The study protocol was reviewed and approved by the Institutional Ethics Committee (IEC), Government Medical College and Hospital, Sector 32, Chandigarh (Approval No. GMCH/IEC/2023/88, dated July 3, 2023). Informed consent was obtained from all participants.

Demographic and clinical data were collected for all enrolled patients. Sputum samples were processed in a Biosafety Level 2 laboratory using a Class II A2 biosafety cabinet. The samples received were divided into three parts: one for acid-fast staining (0.05 mL), the second for culture on Lowenstein-Jensen (LJ) medium (1 mL), and the third for molecular testing using GeneXpert (1 mL) and TB-LAMP (0.06 mL). All tests were performed in parallel. Smears were prepared and heat-fixed, followed by staining using the ZN method. For fluorescent microscopy, smears were prepared and stained with 0.1% auramine solution, followed by decolorization and counterstaining. Brilliant green fluorescence of tubercle bacilli was observed under a fluorescent microscope. For culture, sputum samples were decontaminated using the N-acetyl-L-cysteine-sodium hydroxide (NaOH) method. As soon as rough, tough, and buff colonies were evident, *M. tuberculosis* was confirmed using ZN staining and the MPT64 antigen kit.

Loop-mediated isothermal amplification assay

All steps were performed according to the kit literature. Respiratory samples from patients and the negative control provided in the *M. tuberculosis* complex (MTBC) detection kit were used. Sample lysis followed by DNA extraction was performed using DNA extraction kits. DNA extraction was performed according to the kit literature using the LoopampTM PURE DNA Extraction Kit (Eiken Chemical Co., Ltd., Tokyo, Japan), which has primers targeting gyrB and IS6110. A volume of 60 μL of the sample was dispensed into the heating tube. The heating tube was inverted three to five times to mix the contents and was then flicked down to collect the contents at the bottom. The heating tube was then placed into a dry heating block preheated to 90°C for 5 minutes. After incubation, the heating tubes were removed and allowed to cool to room temperature for 2 minutes and were then inverted three to five times to ensure proper mixing of the contents. Adsorbent tubes were taken and tapped to loosen the white powder, and their caps were removed. The heating tubes were then connected to the adsorbent tubes by screwing, and the solution was released into the adsorbent tubes. The contents were mixed thoroughly. The nozzle of the injection cap was then attached to the adsorbent tube by holding the tube upside down. The injection cap was pushed once and then screwed into place. Amplification was performed according to the kit literature using the LoopampTM MTBC Detection Kit (Eiken, Japan). A volume of 30 μL of extracted DNA from respiratory samples and the negative control was dispensed using a dropper into separate reaction tubes provided in the MTBC detection kit. Similarly, 30 μL of positive control was dispensed into a reaction tube. All reaction tubes were kept inverted for 2 minutes to collect the DNA in the cap and reconstitute the dried reagents present in the cap. The tubes were then flicked down to collect the solution at the bottom of the tube. After confirming that the temperature of the heating block was 67°C, the reaction tubes were loaded into the heating block, and the reaction was started, taking approximately 40 minutes to complete. After 40 minutes, the polymerase enzyme was inactivated by raising the temperature of the heating block to 80°C for 5 minutes, after which the reaction was terminated. Visual detection was performed directly from the reaction tubes under fluorescent light. The reaction tubes were then discarded without opening. Positive results showed light green fluorescence, whereas negative results showed no fluorescence (Figure 1).

**Figure 1:**
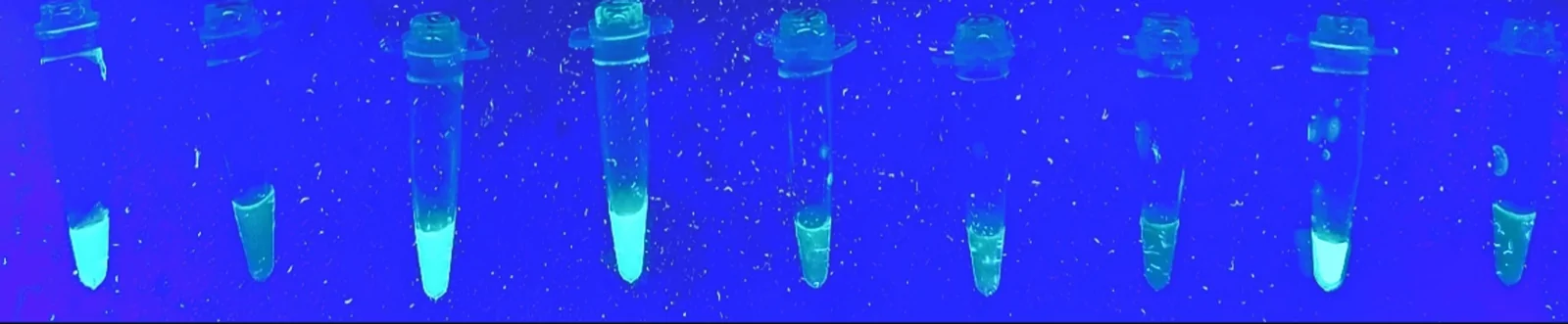
Visual detection of TB-LAMP products under UV light. Bright green fluorescence indicates positive reactions, whereas the absence of fluorescence indicates negative reactions TB-LAMP, tuberculosis loop-mediated isothermal amplification

The GeneXpert MTB/RIF assay (Cepheid, Sunnyvale, CA, USA) was performed according to the manufacturer's instructions. Sample reagent buffer was added to the treated sample, which was then transferred to the Xpert cartridge and loaded into the GeneXpert instrument. Automated processes included specimen filtration, ultrasonic lysis, DNA release, and amplification. Results were interpreted using automated software and categorized as "*M. tuberculosis* detected" or "*M. tuberculosis* not detected," with further classification of rifampicin resistance (RR) based on rpoB gene mutations.

Using the CRS as the reference standard for TB diagnosis, the sensitivity, specificity, positive predictive value (PPV), and negative predictive value (NPV) of the TB-LAMP assay were calculated, along with the corresponding 95% confidence intervals (CIs). The CRS for TB diagnosis included a combination of clinical, radiological, and microbiological criteria: GeneXpert positive and/or mycobacterial culture positive and/or ZN smear microscopy positive and/or clinico-radiological confirmation. The CRS demonstrates a better capacity to identify TB patients compared with culture alone. The use of the CRS was guided by previous evidence indicating the limited efficacy of culture in diagnosing paucibacillary TB. This limitation may consequently lead to underestimation of the sensitivity and specificity of newer nucleic acid amplification tests (NAATs), which may have improved performance in detecting paucibacillary TB cases [8].

Data were analyzed using IBM SPSS Statistics for Windows, version 26.0 (IBM Corp., Armonk, NY, USA). Categorical variables were expressed as frequencies and percentages, whereas continuous variables were summarized as mean ± standard deviation (SD). Diagnostic values of ZN staining, fluorescent microscopy, Xpert MTB/RIF, and TB-LAMP were calculated in terms of sensitivity, specificity, PPV, NPV, and overall diagnostic accuracy, using the CRS as the reference standard. Associations between categorical variables were assessed using the chi-square test. Agreement between TB-LAMP and GeneXpert was assessed using Cohen's kappa coefficient. McNemar's test was used to compare paired proportions of positive results between TB-LAMP and GeneXpert. A p-value of <0.05 was considered statistically significant.

## Results

The study included 100 patients with suspected PTB. The mean age of the study population was 47.45±16.61 years. Of these, 39 (39%) patients were classified as PTB cases and 61 (61%) as non-TB patients according to the CRS. In the TB group, there were 23 (58.97%) males and 16 (41.03%) females.

Acid-fast bacilli (AFB) microscopy yielded positive results in 16 (16%) cases, culture in 25 (25%) cases, GeneXpert identified 34 (34%) positive cases, and TB-LAMP identified 29 (29%) positive cases among the 100 study participants.

The three diagnostic modalities, culture, GeneXpert, and TB-LAMP, were positive in 23 (23%) patients (Figure 2). In contrast, six patients tested positive by both TB-LAMP and GeneXpert, but culture showed no growth of *M. tuberculosis.* GeneXpert alone identified four cases, whereas culture alone and the combination of culture and GeneXpert identified one case each. TB-LAMP did not identify any cases exclusively (Figure 2). Compared with AFB microscopy, culture identified nine additional cases, TB-LAMP identified 13 additional cases, and GeneXpert identified 18 additional cases.

**Figure 2:**
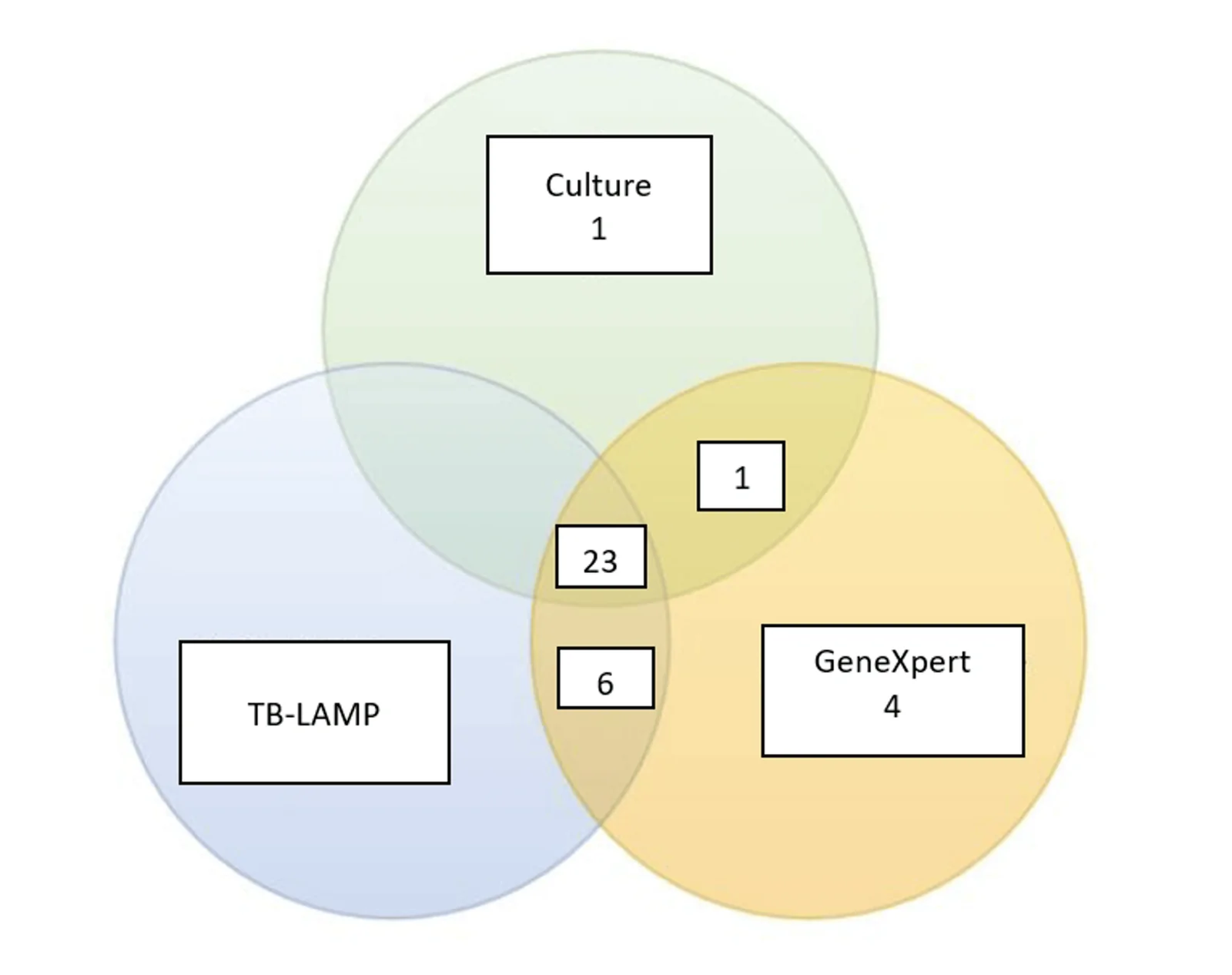
Venn diagram showing the proportion of patients diagnosed by each test and by combinations of tests TB-LAMP, tuberculosis loop-mediated isothermal amplification

The comparative analysis of the tests using the CRS as the reference standard is summarized in Table 1. GeneXpert exhibited the highest sensitivity (87.18%) and specificity (100%), followed by TB-LAMP and culture, whereas AFB microscopy had the lowest sensitivity (41.03%). GeneXpert demonstrated a diagnostic accuracy of 95.0%, followed by culture and TB-LAMP (Table 1).

**Table 1:** Comparison of various diagnostic techniques taking CRS (n=39) as a reference standard CRS, composite reference standard; TB-LAMP, tuberculosis loop-mediated isothermal amplification; CI, confidence interval; AFB, acid-fast bacilli

Test	Sensitivity (%) (95% CI)	Specificity (%) (95% CI)	Positive predictive value (%) (95% CI)	Negative predictive value (%) (95% CI)	Diagnostic accuracy (%) (95% CI)
AFB microscopy	41.03 (25.6 to 57.9)	100 (94.1 to 100)	100 (79.4 to 100)	72.62 (64.3 to 79.5)	77.0 (67.6 to 84.8)
Culture	64.10 (47.2 to 78.8)	100 (94.1 to 100)	100 (86.7 to 100)	81.33 (72.3 to 88.0)	86.0 (77.6 to 92.1)
GeneXpert	87.18 (72.6 to 95.7)	100 (94.1 to 100)	100 (89.7 to 100)	92.42 (83.9 to 97.0)	95.0 (88.7 to 98.4)
TB-LAMP	74.36 (57.9 to 86.9)	91.80 (81.9 to 97.3)	85.29 (68.2 to 95.0)	84.85 (73.9 to 91.9)	85.0 (76.5 to 91.4)

Using culture as the reference standard, the diagnostic performance of the tests is shown in Table 2. When culture served as the reference standard, GeneXpert exhibited the highest sensitivity, followed by TB-LAMP. AFB microscopy exhibited low sensitivity (64.0%). TB-LAMP exhibited a specificity of 85.33%, compared with 86.7% for GeneXpert. The PPV and NPV of TB-LAMP were 67.65% and 96.97%, respectively. The overall diagnostic accuracy of TB-LAMP compared with culture was 87.0% (Table 2).

**Table 2:** Comparison of various diagnostic techniques taking Culture (n=25) as a reference standard TB-LAMP, tuberculosis loop-mediated isothermal amplification; AFB, acid-fast bacilli; CI, confidence interval

Diagnostic test	Sensitivity (%) (95% CI)	Specificity (%) (95% CI)	Positive predictive value (%) (95% CI)	Negative predictive value (%) (95% CI)	Diagnostic accuracy (%) (95% CI)
AFB microscopy	64.0 (42.5 to 82.0)	100 (95.2 to 100)	100 (79.4 to 100)	89.3 (80.4 to 94.6)	91.0 (83.6 to 95.8)
GeneXpert	96.0 (79.6 to 99.9)	86.7 (76.8 to 93.4)	70.6 (53.8 to 83.2)	98.5 (91.7 to 99.9)	89.0 (81.2 to 94.4)
TB-LAMP	92.0 (74.0 to 99.0)	85.33 (75.27 to 92.44)	67.65 (54.48 to 78.51)	96.97 (89.41 to 99.18)	87 (78.80 to 92.89)

The agreement analysis revealed significant concordance between TB-LAMP and GeneXpert. The concordance between the two molecular assays was 90.0%. The Cohen's kappa coefficient was 0.76, indicating substantial agreement. McNemar's test indicated no statistically significant difference in positive rates between the two assays (p=1.00) (Table 3). Of the 34 GeneXpert-positive samples, RR was detected in five cases, whereas TB-LAMP did not detect RR.

**Table 3:** Comparison of TB-LAMP with GeneXpert TB-LAMP, tuberculosis loop-mediated isothermal amplification

TB-LAMP	GeneXpert positive	GeneXpert negative	Total
Positive	29	5	34
Negative	5	61	66
Total	34	66	100

## Discussion

Tuberculosis continues to be a significant public health issue despite considerable progress in diagnostic technology. Timely identification and prompt initiation of treatment are crucial elements of the WHO End TB Strategy, highlighting the need for rapid, sensitive, and decentralized diagnostic techniques. LAMP is a rapid and economical technique that amplifies the target genes gyrB and IS6110 in less than one hour under isothermal conditions. The modality utilizes six primer sets, obviating the need for the sophisticated procedures required for other molecular techniques. The closed-tube system minimizes contamination risks and enables visual detection through fluorescence or turbidity [1,3,9]. In 2016, the WHO endorsed the use of the TB-LAMP assay for detecting MTBC in sputum as a substitute for smear microscopy and as a supplementary test following negative smear microscopy results [10].

Our study revealed that TB-LAMP exhibited a sensitivity of 74.36% and a specificity of 91.80% when assessed against the CRS. The findings align with WHO policy recommendations on TB-LAMP, which indicated pooled sensitivities of 74.1% to 78.0% and specificities of 98.2% to 98.9% compared with culture [11]. Nagai et al. conducted a meta-analysis of 26 trials encompassing 9,330 sputum specimens, revealing pooled sensitivity and specificity of 80.9% and 96.5%, respectively, for the commercial Loopamp MTBC assay [12].

Using culture as the reference standard, TB-LAMP exhibited a sensitivity and specificity of 92.0% and 85.33%, respectively. The results are concordant with the study conducted by Yadav et al., which reported a specificity of 96.8% for TB-LAMP and noted that TB-LAMP significantly outperformed smear microscopy. These findings suggest that TB-LAMP might function as an adjunct or alternative to smear microscopy in peripheral laboratories [13].

In our study, TB-LAMP showed a sensitivity of 74.36% and a specificity of 91.80% against the CRS. Nakiyingi et al. reported a lower sensitivity of 55.4% and a specificity of 98.0% for TB-LAMP in a high TB/human Immunodeficiency virus (HIV)-burden rural setting in Uganda. The lower sensitivity in the Ugandan study may be due to variations in the underlying study population, including a higher proportion of HIV-associated and paucibacillary disease, as well as variations in specimen quality and disease spectrum [14]. Similarly, Nguyen et al. from Vietnam reported a sensitivity of 45.5% and a specificity of 95.1% for smear microscopy [15]. They observed that test performance was markedly affected by the quality and viscosity of the sputum. Gelaw et al. from Ethiopia reported sensitivity and specificity values of 75% and 98%, respectively, which align with the findings of our study [16]. The discrepancies among studies likely reflect variations in patient demographics, prevalence of smear-negative disease, HIV co-infection, specimen quality, and laboratory proficiency.

The efficacy of TB-LAMP in smear-negative cases has received significant attention. Mitarai et al. reported a sensitivity of 55.6% in smear-negative, culture-positive specimens and 98.2% in smear-positive, culture-positive specimens [17]. In our study, TB-LAMP exhibited superior overall performance compared with smear microscopy. Notably, TB-LAMP successfully detected *M. tuberculosis* in 13 of 23 samples that were smear-negative. Kim et al. and Pham et al. reported analogous findings, noting sensitivities ranging from 46.6% to 58.8% in smear-negative cases [7,18]. In our study, AFB microscopy exhibited a sensitivity of 41.03%, while TB-LAMP attained a higher sensitivity of 74.36%. In a multicenter study conducted at microscopy facilities in Lima, Peru; Dhaka, Bangladesh; and Mbeya, Tanzania, Boehme et al. reported an overall specificity of 99.0% (500/505) among culture-negative specimens, a sensitivity of 97.7% (173/177) for TB-LAMP in smear-positive, culture-positive sputum specimens, and 48.8% (21/43) in smear-negative, culture-positive specimens [19]. These results underscore the higher diagnostic performance of TB-LAMP relative to smear microscopy, especially in cases of paucibacillary disease. Conventional microscopy requires approximately 10,000 bacilli/mL of sputum, thereby exhibiting suboptimal performance in paucibacillary cases and early-stage infection [5].

Culture showed a sensitivity and specificity of 64.10% and 100%, respectively, against the CRS. Culture is the microbiological reference standard because of its high specificity and capacity to identify species and assess drug susceptibility; however, in our study, it was less sensitive than TB-LAMP and GeneXpert.

In our study, GeneXpert showed a sensitivity of 87.18% and a specificity of 100% against the CRS. When culture was used as the reference standard, its sensitivity and specificity were 96.0% and 86.7%, respectively. These results align with the initial multicenter study conducted by Helb et al., in which GeneXpert identified 29/29 (100%) smear-positive, culture-positive cases and 38/53 (71.7%) smear-negative, culture-positive cases in Vietnam, with 100% specificity among culture-negative specimens; in a Uganda cohort, it identified 63/64 (98.4%) culture-positive cases [20]. The higher sensitivity of GeneXpert may be attributed to its ability to detect low bacterial loads in real time, along with its ability to detect RR. RR was detected in five of 34 (14.71%) GeneXpert-positive cases and five of 39 (12.82%) CRS-confirmed TB cases in our study. Fulwani et al. found similar results in a tertiary-care hospital in Jaipur, Rajasthan, where 10% of 100 patients with newly diagnosed PTB had RR [21].

Analysis of agreement revealed significant concordance between TB-LAMP and GeneXpert, with an overall agreement of 90% and a kappa coefficient of 0.76. Moreover, McNemar's test revealed no statistically significant difference in the positive rates of the two assays. These results align with the findings of Nagai et al. and Shete et al., who reported similar diagnostic performance of TB-LAMP and GeneXpert [12,22]. While GeneXpert is superior due to its higher sensitivity and capacity to identify RR, the difference in diagnostic performance between the two assays was relatively small in our study. The overlap analysis of microbiologically confirmed cases highlights the complementary role of molecular diagnostics and suggests that molecular techniques may improve tuberculosis detection compared with traditional culture and smear microscopy.

The WHO End TB Strategy emphasizes early diagnosis and treatment. Delays in tuberculosis diagnosis can prolong transmission and worsen treatment outcomes. Thus, all levels of the healthcare system need rapid, accurate, and accessible diagnostic technologies to achieve the End TB Strategy [23,24]. In our study, TB-LAMP had higher sensitivity than smear microscopy, with diagnostic performance approaching that of GeneXpert. The assay had high specificity, rapid turnaround, and good agreement with GeneXpert. Programmatically, TB-LAMP has several advantages over GeneXpert, including one-hour testing, less need for sophisticated infrastructure and training, and ease of implementation. The use of TB-LAMP at peripheral and district-level laboratories could increase early case detection, reduce diagnostic delays, and aid national tuberculosis elimination efforts.

This study had some limitations. This was a single-center study with a relatively small sample size, potentially constraining the precision of the diagnostic accuracy estimates. Comprehensive subgroup analyses of TB-LAMP efficacy among smear-negative, HIV-positive, and previously treated tuberculosis patients were not conducted because of insufficient sample sizes. This necessitates further studies through large multicenter research.

## Conclusions

TB-LAMP demonstrated much higher sensitivity compared with conventional smear microscopy, with diagnostic performance approaching that of GeneXpert. Nevertheless, the reported sensitivity and specificity of TB-LAMP were lower than those of GeneXpert. In addition, RR could not be inferred from TB-LAMP. The observed agreement between TB-LAMP and GeneXpert should not be interpreted as evidence of diagnostic equivalence or interchangeability.

TB-LAMP may have potential utility as an adjunct to or alternative to smear microscopy, especially in decentralized and resource-constrained healthcare settings. Nonetheless, larger multicenter studies are required to validate these findings and determine the appropriate role of TB-LAMP within existing tuberculosis diagnostic algorithms.
